# Sleep Restriction during Simulated Wildfire Suppression: Effect on Physical Task Performance

**DOI:** 10.1371/journal.pone.0115329

**Published:** 2015-01-23

**Authors:** Grace Vincent, Sally A. Ferguson, Jacqueline Tran, Brianna Larsen, Alexander Wolkow, Brad Aisbett

**Affiliations:** 1 Centre for Physical Activity and Nutrition Research, Deakin University, Burwood, 3125, Australia; 2 Bushfire Co-Operative Research Centre, East Melbourne, 3002, Australia; 3 Central Queensland University, Appleton Institute, Wayville, 5034, Australia; 4 Centre for Exercise and Sport Science, Deakin University, Burwood, 3125, Australia; University of Pennsylvania Perelman School of Medicine, UNITED STATES

## Abstract

**Objectives:**

To examine the effects of sleep restriction on firefighters’ physical task performance during simulated wildfire suppression.

**Methods:**

Thirty-five firefighters were matched and randomly allocated to either a control condition (8-hour sleep opportunity, n = 18) or a sleep restricted condition (4-hour sleep opportunity, n = 17). Performance on physical work tasks was evaluated across three days. In addition, heart rate, core temperature, and worker activity were measured continuously. Rate of perceived and exertion and effort sensation were evaluated during the physical work periods.

**Results:**

There were no differences between the sleep-restricted and control groups in firefighters’ task performance, heart rate, core temperature, or perceptual responses during self-paced simulated firefighting work tasks. However, the sleep-restricted group were less active during periods of non-physical work compared to the control group.

**Conclusions:**

Under self-paced work conditions, 4 h of sleep restriction did not adversely affect firefighters’ performance on physical work tasks. However, the sleep-restricted group were less physically active throughout the simulation. This may indicate that sleep-restricted participants adapted their behaviour to conserve effort during rest periods, to subsequently ensure they were able to maintain performance during the firefighter work tasks. This work contributes new knowledge to inform fire agencies of firefighters’ operational capabilities when their sleep is restricted during multi-day wildfire events. The work also highlights the need for further research to explore how sleep restriction affects physical performance during tasks of varying duration, intensity, and complexity.

## Introduction

Australian firefighters regularly work shifts of up to 15 h per day over several days during wildfire suppression [1,2]. Wildfire incidents are often long in duration, and certain deployments may require firefighters to travel considerable distances from their home location. During such deployments, fire personnel sleep in temporary accommodation near the fireground between consecutive shifts [3]. Fireground conditions such as heat, light, smoke, noise, and unfamiliar surroundings may contribute to an inadequate sleeping environment, which may compromise sleep quantity and quality [1,4]. Accordingly, firefighters have subjectively reported obtaining 3–6 h sleep per night during wildfire suppression deployments [1,5]. Elements of shift work such as night work, long working hours, and consecutive shifts have all been associated with an increased risk of accidents [6–8]. While the effects of multiple days of sleep restriction on cognitive function are well established [9,10], less is known about its effects on physical task performance. In response to an emergency event, wildfire personnel must perform physical work [11]. If task performance and/or the underlying physiology are compromised due to poor sleep, this may adversely impact firefighters’ health and safety and the collective emergency response.

Although there is a lack of robust empirical data detailing the effects of sleep restriction on firefighters’ physical work performance, insights can be gained from sustained military operations [12–14] and laboratory studies [15,16]. Early military studies assessed physical performance following periods of sleep restriction ranging from 3.0–5.3 h per night, over 5–9 days. Preservation of self-paced work output but decrements in upper body 30 s mean power were observed during simulated combat scenarios [12–14]. Others have observed that physical work output involving short-term maximal efforts was maintained under periods of complete sleep deprivation [15,16]. Yet in contrast, declines in self-paced work rate have been reported in low- to moderate-intensity tasks performed during 48 h of total sleep deprivation [16]. This decline was attributed to reduced motivation levels due to either task repetition in a laboratory environment, an increase in perceived exertion, or a combination of both factors [16]. To our knowledge, no study has examined the effect of sleep restriction on the performance of intermittent, variable-intensity manual handling work over multiple days, which is typical of wildland firefighting [11].

The aforementioned military and laboratory studies have employed experimental protocols with varying designs. In particular, the duration of the sleep restriction period, work parameters (task type, intensity and duration), and calorie restriction all differ between studies. This makes it difficult to accurately quantify the effect of sleep restriction on worker physical task performance and physiology and to extrapolate these findings to wildfire personnel. Moreover, implementing controlled field studies is not often feasible as wildfire conditions can be dangerous to both firefighters and researchers. Wildfire conditions such as heat, smoke, and varying terrain are additional confounders which may make it challenging to accurately determine the influence of sleep restriction on physical task performance. In addition, the work performed by personnel within this dynamic environment is variable [17] and therefore not standardised within- or between-individuals across a multi-day wildfire. Therefore, the aim of the current study was to determine the effect of sleep restriction on firefighters’ physical task performance, physiology, and perceptual responses during simulated multi-day wildfire suppression. This study employed a rigorously controlled laboratory protocol implementing a valid work task simulation [18] to quantify the effects of sleep restriction on physical performance.

## Materials and Methods

### Participants and screening

Thirty-five volunteer and career firefighters (30 males, 5 females) were recruited, from Australia’s state fire agencies (Victoria, South Australia, New South Wales, Tasmania). The sample size was estimated by a magnitude based-statistical power analysis [19] using an averaged effect size of 0.3 ± 0.2 from relevant firefighting research [20,21] and an α = 0.05 and β = 0.80. Individual participants were matched, in order of priority, by sex, age, and body mass index to reduce variation between conditions and then were randomly allocated to either the control condition (CON; n = 18) or the sleep-restricted condition (SR; n = 17). Participants provided written informed consent, completed a general health questionnaire [22] to ensure they were able to complete vigorous exercise without medically supervised exercise testing, and were screened for diagnosed sleep disorders. Ethical approval was obtained from the Deakin University Human Research Ethics Committee and the Human Research Ethics Committee of CQUniversity, and written informed consent was provided by all participants before the commencement of the study.

Participants were instructed to maintain their normal sleep behaviour prior to entering the study. Participants wore an activity monitor for two days pre-simulation (*Actical* MiniMitter/Respironics, Bend, OR) on their non-dominant wrist and completed a sleep diary to evaluate their pre-simulation sleep hours and timing of sleep periods. Activity monitors were set to sample in 1-min epochs, with a sensitivity of < 40 counts per epoch to distinguish between sleep and wake states [23].

Participants’ age, height, and weight were recorded prior to testing. Height was measured without shoes using a stadiometer (Fitness Assist, Wrexham, England). Semi-nude body mass was measured using an electronic scale (A and D, Japan). Participants wore their own firefighting personal protective clothing throughout the simulation. This included a two-piece jacket and trouser set made from Proban cotton fabric (Protex, Australia), suspenders, boots, gloves, helmet, and goggles (amounting to ~5 kg). The participant characteristics of the CON and SR conditions are shown in Table 1.

**Table 1:** Characteristics of firefighters in the control and sleep restricted conditions.

	**CON**	**SR**
*n*	18	17
Age (y)	39 ± 16	39 ± 15
Body mass (kg)	85.1 ± 17.7	93.8 ± 20.2
Height (m)	1.78 ± 0.08	1.78 ± 0.07
BMI (kg·m^−2^)	26.7 ± 4.8	29.6 ± 5.5
Service (y)	9 ± 9	10 ± 6
Male:Female	15:3	15:2

Values are in mean ± SD; BMI, body mass index.

### Experimental protocol

Participants were required to attend the laboratory for four days and the study condition (CON or SR) was blinded from the participants prior to arrival. Participants arrived at the testing facility at 6:00 p.m. on the pre-study day and were familiarised with all daily procedures. Throughout the testing period, participants followed a strict daily schedule including work bouts, meal times, and sleep periods. The day was divided into two-hour work blocks, with each block comprising 55 min of physical work, 20–25 min of physiological testing, 20–25 min of cognitive testing, and a 15–20 min rest period. The suite of cognitive procedures was administered as part of another study and will be reported elsewhere. Participants were instructed that their caffeine consumption and cigarette smoking could continue as it normally would during a wildfire suppression deployment, but that these behaviours were restricted to the rest periods which occurred for 15–20 min within each two-hour period. When compared to habitual caffeine consumption, there were no differences between the groups in caffeine intake across the simulation.

The simulated environment was kept at a moderate temperature (18–20°C) throughout the testing period. Room temperature was maintained through the use of split cycle air-conditioners (Daikin Industries Ltd, Japan). Ambient air temperature was monitored throughout testing using a wireless temperature and humidity data logger (HOBO ZW_003, One Temp Pty.Ltd, Australia), data receiver (HOBO ZW_RCVR, One Temp Pty Ltd, Australia), and associated software (HOBO Pro Software, One Temp Pty Ltd, Australia).

On day one firefighters completed three two-hour work circuits: one familiarisation work block (12:30–2:30 p.m.), then two subsequent work blocks (2:30–4:30 p.m.; 4:30–6:30 p.m.). Day one occurred prior to the sleep intervention providing each individual with a baseline against which all subsequent measures of each outcome variable were compared. Days two and three began at 8:00 a.m. and on both days, the firefighters performed five two-hour work circuits (8:00–10:00 a.m., 10:00 a.m.–12:00 p.m., 12:30–2:30 p.m., 2:30–4:30 p.m. and 4:30–6:30 p.m.). As such, the baseline value was labelled as circuit 0 and every subsequent circuit performed labelled sequentially (circuit 1–10). Breakfast (6:30–7:00 a.m.), lunch (12:00–12:30 p.m.), and dinner (6:30–7:00 p.m.) were at the same time on all three days.

Daily fluid consumption was precisely recorded to monitor participants’ hydration status as hyper- and hypo-hydration can impact physical performance [24]. Participants were able to drink room temperature water ad libitum from marked, supplied bottles. Each day participants were provided with two sachets of carbohydrate supplement which they could add to their water at any time. Breakfast, lunch, and dinner meal items were based on food normally available to firefighters during wildfire suppression. Participants were also provided with a “ration pack” containing a variety of food items similar to those available during wildfire suppression. All meal and snack food items were identified in consultation with subject matter experts from Australasian fire authorities. Types and quantities of ingested food were recorded throughout the protocol. There was no difference in fluid and total energy intake between the groups.

To replicate sleeping conditions during a wildfire suppression deployment [1], participants slept on camp beds in the simulated environment. The pre-study day involved an adaptation night consisting of an 8-h sleep opportunity for both conditions. On day two and three, the CON condition was assigned an 8-h sleep opportunity from 10:00 p.m. to 6:00 a.m. whereas those in the SR condition were assigned a 4-h sleep opportunity from 2:00 a.m. to 6:00 a.m. Both conditions were constantly observed by research personnel to prevent them from falling asleep outside of their designated sleeping period. Between dinner (7:00 p.m.) and the beginning of the sleep opportunity (CON; 10:00 p.m. and SR; 2:00 a.m.) participants engaged in sedentary leisure activities (i.e. read a book, watch a movie).

### The physical work circuit

The firefighting circuit was developed using a job task analysis [11] of wildfire suppression tasks and verified by panels of firefighter subject matter experts [18]. The tasks involved simulated actions, fitness components, and movements performed during wildfire suppression [11,25]. These tasks were chosen on the basis of being the longest, most intense, or most frequent tasks performed during wildfire suppression work [25]. They were also considered the most physically demanding and operationally important [11]. The six physical tasks include: charged hose advance, blackout hose work, hose rolling, lateral repositioning, rake, and static hold.

Five minutes were allocated to each task, with task-specific work-to-rest ratios within each 5-min block in accordance with the work-to-rest ratios observed for each task in live fire suppression conditions [25]. Some tasks were only performed once in each 55-min block, whereas others were performed multiple times according to their recorded frequency during live fire suppression [25]. Participants completed the tasks in an ordered circuit; each participant began the circuit at one of five task stations (charged hose advance, blackout hose work, hose rolling, lateral repositioning, or rake) but rotated through each subsequent task in the same order irrespective of start point. The static hold task was always the last task performed in the circuit and by all participants concurrently. This allowed participants to perform some tasks at the same time. CON and SR participants were matched on age, then sex, and body mass index and then randomly allocated into the five circuit start points. This process prevented potential clustering of specific demographic variables into any one of the five circuit start points. Each participant began at the same individually-assigned start point during all physical work bouts. Task performance was evaluated for each 5-min work block completed for each task, as discussed in further detail below.

#### Charged hose advance

Each participant dragged a 2-m rubber hose (38-mm diameter, with branch) attached to a 15-kg weighted tyre up and back for 8 m along a carpeted surface, marked at 2-m increments. The hose was filled with rice to simulate a charged hose (i.e., pressurised with water). This task simulates forward movement with a charged hose towards the fire front [11]. This task was undertaken with a work-to-rest ratio of 65 s work to 55 s rest, which was completed twice within a 5-min period, with one 5-min work bout performed during the 55-min circuit. The participant was instructed to stop completely at the end of each work period, at which point task performance was recorded as distance covered (to the nearest 2-m marker).

#### Blackout hose work

Each participant dragged a 2-m rice-filled rubber hose (38-mm diameter, with branch), attached to a 15-kg weight bag around a 2.5-m × 2.5-m square, stopping at each corner for a period of 3 s timed by a metronome. This task simulated the stop start movements firefighters perform when extinguishing smouldering debris, during post-fire clean up [11]. This task was undertaken with a work-to-rest ratio of 90 s work to 60 s rest, which was completed twice within the 5-min period, with two 5-min work bouts performed during the 55-min circuit. The participants walked clockwise during the first work bout and anticlockwise during the second work bout to incorporate both left and right manoeuvres. The participants were instructed to stop at the end of each work period where their total distance was recorded to the nearest corner of the square.

#### Hose rolling

The participants were instructed to roll-up a 8-m rubber hose (38-mm diameter, 16-m length hose folded in half, with branch) beginning at the folded end of the hose and moving along the length of the hose rather than pulling the hose towards them. This task simulated rolling up a hose and therefore had to be rolled to operational standard (a tight coil with edges aligned). This task was undertaken with a work-to-rest ratio of 60 s work to 60 s rest, which was completed twice during the 5-min period, with one 5-min work bout performed during the 55-min circuit. The hose was taped 0-, 2-, 4-, 6-, and 8-m intervals to aid in recording distance.

#### Lateral repositioning

The participant walked the arc (11 m) of a 3.5-m radius semi-circle carrying a 3.5-m rice filled rubber hose (38-mm diameter, with branch). The hose was anchored by a chain to a stand positioned in the middle of the semi-circle allowing the hose to freely rotate. Two platforms (68 × 28 × 15 cm) were positioned at ¼ and ¾ distance markers of the semicircular arc, with one platform placed length ways and the other placed cross ways. Participants walked over the step placed cross ways and stepped up with two feet onto and then off the step placed length ways. This task simulated walking with a charged hose over obstacles such as tree roots and other debris commonly found on the fireground [11]. This task was undertaken with a work-to-rest ratio of 30 s work to 30 s rest, which was completed four times during the 5-min period, with four 5-min work bouts performed during the 55-min circuit. Task performance was measured as distance travelled during each work period, rounded to the nearest quarter (2.75 m) completed.

#### Rake

Participants were instructed to rake the contents of a 2-m × 0.9-m box filled with 29 kg of 1-cm tyre crumb and large tyre pieces. The tyre crumb simulated ground debris that is cleared when creating a mineral earth fire break [11]. Two identical boxes were placed side by side and participants alternated between the boxes after successfully raking the contents from one side to the other. Participants utilised a 35-cm dual-sided rakehoe (Cyclone Industries, Australia) to complete this task. This task was undertaken with a work-to-rest ratio of 90 s work to 60 s rest, which was completed twice during the 5-min period, with one 5-min work bout performed during the 55-min circuit. Task performance was measured by the area of material moved (m^2^) (i.e., the amount of material moved from one side of the box to the other).

#### Static hold

The participant was instructed to hold a 3.5-m rice-filled rubber hose (38-mm diameter, with branch) attached to a stand with a looped elasticised rope providing resistance when held off the ground. A laser was positioned at the end of the hose so that participants could aim the hose towards a target placed 1.5 m above the ground. This task simulated holding a charged hose to direct water or other extinguishing material towards a fire, while stationary [11]. The participants could hold the hose at their waist or over their shoulder with their chosen front foot placed completely over the line marked at 4.8 m from the centre point of the hose stand. The participants were instructed to hold the hose for the entire 5-min period. If a participant dropped the hose, the laser went out of the target for more than 2 s, or the participant could not hold the position for 5 min, the failure time was recorded. This task was performed once during the 55-min circuit.

### Heart rate

Daily heart rate was recorded using the Team Polar (Polar Team^2^, Kempele, Finland) heart rate system from 6:30 a.m. to 6:00 p.m. on all testing days. Heart rate was logged every 5 s, with data downloaded daily and analysed using the Polar Team 2 Pro Software (Polar, Kempele, Finland). Relative average and peak heart rate was predicted using HR_max_ = 207 − (0.7 × age) [26] to individualise the cardiovascular response due to the large spread in age (18–61 years) across the sample. Average and peak heart rates are analysed during the physical work circuit, the rest period, and during each physical task performed.

### Core temperature

Participants ingested a core temperature capsule (Jonah, Minimitter, Oregon) prior to sleep each evening (9:30 pm), in order to allow adequate time for the capsule to pass through the stomach to the small intestines (Lee et al., 2000). Intestinal temperature (via ingested capsules) was preferred over rectal thermometry, as it is a valid but less invasive index of core temperature [27]. Core temperature was recorded continuously on a data logger (VitalSense, Minimitter, Bend, Oregon) throughout the testing period.

### Rating of perceived exertion and effort sensation scale

Participants were asked to report a rating of perceived exertion (RPE, on a scale of 6–20; Borg, 1982) [28][28] after each work bout (i.e., every 5 min). After the completion of each physical work circuit participants were asked to report their physical effort using a modified Borg scale where effort < 100% is reported [29]. Participants were asked ‘how much of yourself did you give’? Items ranged from 0% ‘gave no effort at all’ to 100% ‘gave absolutely everything, nothing left’.

### Worker activity

An activity monitor (*Actical* MiniMitter/Respironics, Bend, OR) was worn on the participants’ non-dominant wrist (dominance was defined as the participants’ preferred writing hand) throughout the simulation to assess activity. Whole body motion in a three dimensional plane was measured at 1-min intervals. Data was downloaded using Actical software (version 3.10 MiniMitter/Respironics, Bend, OR) and expressed as absolute counts.

### Sleep monitoring: Polysomnography

Polysomnography (PSG) was utilised during the four study nights to assess sleep architecture [30,31]. PSG arrangement and recording began each night at 9:00 p.m. for both conditions. Standard PSG equipment (Compumedics E Series, Melbourne) was arranged as follows: EEG (Oz and Cz positions); EOG (outer canthi of each eye); EMG (masseter and facial muscles); the earth electrode on the right clavicle. All signals were recorded using gold Grass electrodes with initial impedances below 10 kΩ. The PSG recordings were scored according to standard criteria of AASM [32] in 30-s epochs. Each epoch was assigned a stage of sleep (Stages 1–3, REM) or wake by a blinded scorer using Profusion 3 software (Compumedics E Series, Melbourne, Australia). From each sleep period, participants’ total sleep time was calculated. Participants were continuously monitored throughout the study to ensure napping did not occur.

### Statistical analyses

All statistical analyses were carried out using Stata 12.0 (StataCorp, Texas, USA). Exploratory data analysis was conducted to determine whether the data met parametric assumptions of normality and homoscedasticity. Each individual’s performance scores were normalised to reflect changes from their individual specific baseline (as previously mentioned, pre-intervention task performance on day one of the simulation). Participant characteristics, total sleep hours, energy intake, hydration status, and caffeine consumption were normally distributed, thus one-way analyses of variance (ANOVA) were used to determine between-group differences. For all other variables, generalised linear mixed models (GLMMs) were constructed using the Generalized Linear Latent and Mixed Model software (*gllamm;* version 2.3.20). This modelling procedure has previously been employed in sleep literature [9,33] and is increasingly preferred over traditional repeated-measures ANOVA, as GLMMs better account for the serial correlation of data points over time [34]. The *gllamm* also provides valid estimates in the presence of missing data, and uses maximum likelihood estimation with adaptive quadrature for more reliable parameter estimates than adaptive quadrature [35]. Readers are encouraged to seek out the comprehensive reviews of these procedures by Rabe-Hesketh [35,36], and a detailed comparison of linear mixed models and ANOVAs in sleep research [37].

Mixed effects models incorporate *fixed* effects that determine the influence of the experimental conditions (e.g., assignment to a CON or SR condition), alongside *random* effects that considers each individual as having a unique response to the intervention (e.g., inter-individual differences in response to periods of sleep restriction) [38,39]. Therefore, mixed models enable the distinction of between-subject (i.e, inter-individual) from within-subject (i.e, intra-individual) effects. The framework recommended by Singer [40] guided the construction of mixed models to iteratively investigate the fixed effects of Condition, Circuit, and the interaction of Condition × Circuit, with random intercepts and random slopes that varied at the Participant-level. All dependent variables exhibited a Gaussian distribution, thus the identity link function was specified [41]. The CON and SR conditions were coded 0 and 1, respectively. Therefore, a positive β value for the effect of Condition indicates CON > SR and a negative β value indicates SR > CON. For Circuit effects, a positive β value indicates an increase over successive circuits and a negative β value indicates a decrease. The random effects express the variance due to the inter-individual differences at baseline (random intercept) and over time (random effect of Circuit). Selection of the optimal model for each outcome variable was informed by comparing Akaike weights between candidate models as per the procedure outlined by [42]. If the Akaike weights between two competing models had similar probabilities of being the best model, the model with the fewest number of parameters was preferred in accordance with the principle of parsimony. The final parameter estimates are reported in-text as β coefficient ± standard error of the estimate (SE), *P* value. Statistical significance was set at *P* ≤ 0.05 and all data are presented as means ± standard deviations unless otherwise stated.

## Results

There was no difference between CON and SR in participants’ age, body mass, body mass index, height, or years of service (*P ≥* 0.110; Table 1). There were no differences in ambient temperature (CON 19.2 ± 1.1°C; SR 19.6 ± 1.6°C; *P* = 0.220) or humidity (CON 55.8 ± 6.3%; SR 55.9 ± 6.8%; *P* = 0.980) between conditions.

### Sleep hours

There were no differences between the two conditions in mean sleep duration obtained in the two days prior to the simulation (*P ≥* 0.283), nor on the pre-study adaptation night (CON 6.3 ± 0.9 h; SR 6.4 ± 0.7 h; *P* = 0.726. During the two experimental nights, mean sleep duration was lower in the SR (3.6 ± 0.3 h) condition compared to CON (6.9 ± 0.4 h; *P <* 0.001).

### Physical task performance

**Figure 1:**
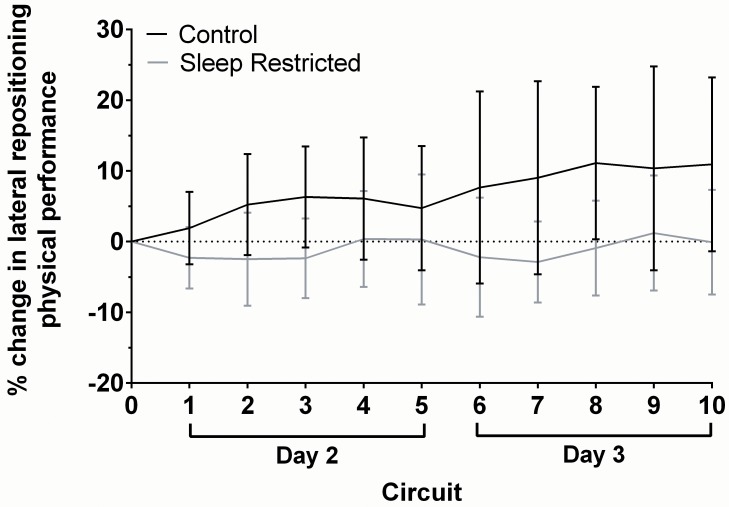
Lateral repositioning task performance during the multi-day simulation. Note the considerable inter-individual variability within both CON and SR groups at each time point, as denoted by the error bars (± 1 SD).

Daily mean performance on each physical task is shown in Table 2. For all physical tasks, the fixed effect of Condition did not significantly improve upon the amount of variance explained by simpler models. Random slope models best explained variances in physical performance (Table 3). Fig. 1 demonstrates the inter-individual variability in lateral repositioning task performance, indicative of the same pattern observed for the other tasks. Participants in both conditions successfully completed the 5-min static hold hose task during every two-hour period throughout the simulation, and therefore this data will not be reported.

**Table 2:** Daily physical task performance during the physical work circuit.

**Task**	**Condition**	**Day 1 (Baseline)**	**Day 2**	**Day 3**
Charged hose advance (m)	CON	107.8 ± 24.1	111.2 ± 26.1	116.6 ± 30.2
	SR	99.9 ± 12.3	104.4 ± 16.1	108.0 ± 17.6
Blackout (m)	CON	169.4 ± 14.5	168.0 ± 15.9	166.8 ± 16.1
	SR	167.9 ± 16.0	167.0 ± 17.1	167.4 ± 18.6
Hose-rolling (m)	CON	18.7 ± 4.8	21.6 ± 5.6	24.8 ± 7.5
	SR	16.9 ± 3.6	19.0 ± 3.9	20.5 ± 5.0
Lateral Repositioning (m)	CON	631.4 ± 92.1	657.9 ± 86.1	689.8 ± 91.1
	SR	650.5 ± 76.7	656.5 ± 76.8	676.7 ± 73.3
Rake (m^2^)	CON	5.0 ± 1.4	5.5 ± 1.5	5.6 ± 1.5
	SR	4.6 ± 0.6	4.9 ± 0.8	5.1 ± 0.8

**Table 3:** Generalised linear mixed model parameter estimates for physical performance variables.

	**Lateral Repositioning**	**Hose rolling**	**Rake**	**Charged hose advance**	**Black out hose**
**Fixed effects**					
*Intercept*	2.05 (1.26)	1.31 (4.50)	8.17 (2.13)	2.71 (1.49)	−0.88 (0.90)
**Random effects**					
*Intercept*	37.40 (12.16)	484.95 (169.75)	115.55 (46.88)	110.03 (9.30)	26.27 (10.49)
*Circuit*	2.14 (0.57)	26.77 (7.36)	7.20 (2.20)	20.54 (17.97)	0.37 (0.20)
*Residual*	19.67 (1.67)	330.06 (27.94)	163.15 (13.83)	3.69 (1.21)	35.39 (3.02)

Data presented as: β parameter estimate (standard error).

### Heart rate and core temperature

There was no effect of Circuit, Condition, or Condition × Circuit for average heart rate (%HR_max_) across the two-hour work circuit (*P ≥* 0.480) or during the physical work component (*P ≥* 0.490) or rest period (*P ≥* 0.270). There was no effect of Circuit, Condition, or Condition × Circuit for peak heart rate (%HR_max_) across the two-hour work circuit (*P* ≥ 0.800) or during the physical work component (*P* ≥ 0.940) or rest period (*P* ≥ 0.420). Heart rate was also analysed for each individual physical task. For almost all tasks, the intercept-only and random slope models best explained variances in average and peak heart rate (Table 4). Peak heart rate during the black out hose task was best explained by the model including a single fixed effect for Circuit (β = −0.21 ± 0.08; *P* < 0.05). For core temperature, the greatest relative likelihood was achieved by the full model with significant fixed effects for Condition (β = −0.15 ± 0.07; *P* < 0.05) and Condition × Circuit (β = 0.02 ± 0.01; *P* < 0.05).

**Table 4:** Generalised linear mixed model parameter estimates for average and peak heart rate for each physical task.

	**Lateral Repositioning**		**Hose rolling**		**Rake**		**Charged hose advance**		**Black out hose**		**Static hold**	
	Average	Peak	Average	Peak	Average	Peak	Average	Peak	Average	Peak	Average	Peak
**Fixed effects**												
*Intercept*	62.80 (1.34)	72.17 (1.62)	64.29 (1.37)	75.62 (1.58)	70.63 (1.29)	80.24 (1.36)	72.64 (0.53)	83.01 (1.75)	62.31 (1.43)	70.26 (1.60)	59.24 (1.46)	66.32 (1.49)
*Circuit*										0.21 (0.08)		
**Random effects**												
*Intercept*	61.91 (15.04)	89.28 (21.85)	67.08 (17.12)	85.64 (22.05)	54.99 (14.27)	57.80 (15.46)	71.17 (6.78)	100.25 (25.61)	65.24 (16.43)	82.58 (20.22)	77.78 (19.99)	80.64 (20.91)
*Circuit*			0.18 (0.08)	0.23 (0.10)	0.28 (0.10)	0.28 (0.11)	0.17 (0.07)	0.22 (0.10)	0.11 (0.05)		0.28 (0.10)	0.40 (0.14)
*Residual*	11.14 (0.84)	23.62 (1.79)	14.24 (1.13)	18.68 (1.49)	13.70 (1.09)	21.10 (1.69)	17.34 (1.36)	21.38 (1.70)	9.96 (0.79)	22.13 (1.67)	13.63 (1.09)	20.80 (1.66)

Data presented as: β parameter estimate (standard error).

### Rating of perceived exertion and effort sensation scale

The intercept-only and random slope models best explained variances in rating of perceived exertion during the lateral repositioning, hose rolling, black out hose, and static hold tasks (Table 5). An interaction of Condition × Circuit was observed for rake and charged hose (Table 5). While this may suggest that those in the control group perceived these tasks to be more difficult over the course of the simulation, the parameter estimates were very small in magnitude and thus require cautious interpretation. The random slope model best explained variance in effort sensation (β = 78.83 ± 1.80; *P* < 0.05).

**Table 5:** Generalised linear mixed model parameter estimates for perceptual measures for each physical task.

	**Lateral Repositioning**	**Hose rolling**	**Rake**	**Charged hose advance**	**Black out hose**	**Static hold**
**Fixed effects**						
*Intercept*	11.39 (0.13)	11.94 (0.19)	13.87 (0.29)	14.08 (0.36)	12.14 (0.15)	12.95 (0.26)
*Condition*			0.29 (0.40)	0.95 (0.50)		
*Circuit*		0.08 (0.01)	0.02 (0.02)	0.06 (0.03)		
*Condition* × *Circuit*			0.08 (0.03)	0.09 (0.04)		
**Random effects**						
*Intercept*	0.61 (0.15)	0.97 (0.25)	1.18 (0.30)	1.76 (0.45)	0.78 (0.20)	2.21 (0.55)
*Residual*	0.25 (0.02)	0.72 (0.05)	0.73 (0.06)	1.23 (0.09)	0.38 (0.03)	0.89 (0.07)

Data presented as: β parameter estimate (standard error).

### Work activity

**Figure 2:**
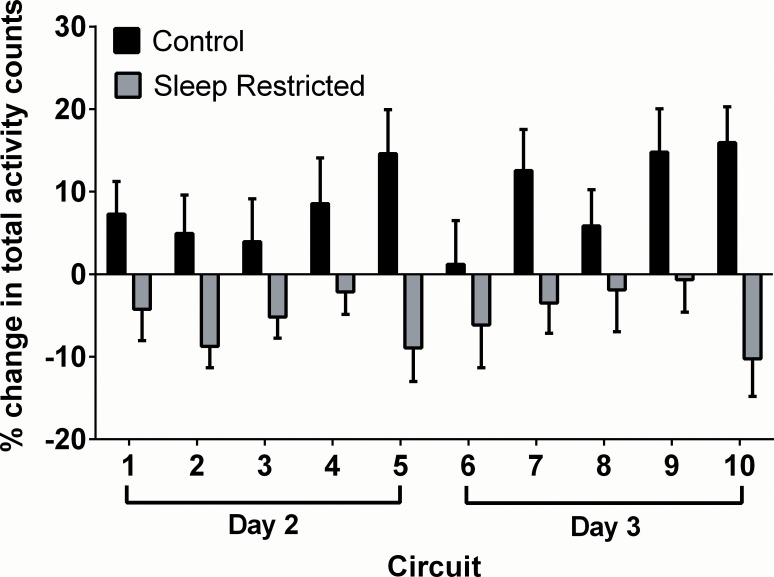
Percentage change in total activity counts relative to baseline across each two-hour work circuit.

Total activity counts were summed for each two-hour work circuit. The two-hour work circuit was also divided into two major components: the physical work circuit and the rest period. The conditional growth model with random slopes best explained variance in activity throughout the two-hour work circuit (fixed effect of Condition: β = 11.92 ± 2.25; *P* < 0.05; random effect of Circuit: β = 1.22 ± 0.58). This was also the case for the physical work circuit (fixed effect of Condition: β = 5.02 ± 1.99; *P* < 0.05; random effect of Circuit: β = 3.24 ± 0.97). A main effect for Condition (β = 20.50 ± 4.34; *P* < 0.001) was also evident during the rest period. These results indicate that the CON participants recorded greater activity during work and rest periods compared to the SR participants, and that activity counts during work periods increased over the course of the simulation. The percentage change in total activity counts relative to baseline across each two-hour work circuit is shown in Fig. 2.

## Discussion

This study examined the effects of 4 hours of sleep restriction on firefighters’ physical task performance, physiology, and perceptual responses during multi-day simulated wildfire suppression. The findings suggest that physical task performance was unaffected by 4 hours of sleep restriction. Heart rate, core temperature, and perceptual responses to these tasks were also largely unaffected by sleep restriction. However, sleep-restricted participants were significantly less active throughout the simulation compared to control participants.

Sleep restriction did not impact upon firefighters’ physical work performance during multi-day simulated wildfire suppression. In addition, sleep restriction had limited effects on the physiological and perceptual measures, further supporting the assertion that there was no real difference in task performance. There were no observable differences between the groups in relative average and peak heart rate throughout the simulation. This finding is consistent with previous work, where resting average heart rate remained unchanged during partial sleep restriction [43]. Moreover, sub-maximal exercising heart rate was also unchanged following total sleep deprivation periods of 36 and 64 h [44,45]. Our findings indicate that core temperature was lower in the control group and decreased for all participants across the simulation. However, the parameter estimates are very small and therefore do not likely reflect a meaningful difference or change. This finding is consistent with previous investigations that have observed either no change [46,47] or a small decrease [48,49] in body temperature following sleep restriction. While the tasks that were included in the simulation were highly representative of those performed during actual fire suppression [11,18], it is possible that these physical tasks were not sensitive enough to detect changes in task performance following sleep restriction; further research is needed to test this assertion. However, the decision to select tasks that may be more sensitive to sleep restriction must be balanced against the intent to maintain a representative design [50], which is important to ensure that experimental observations can be generalised to a real wildfire suppression deployment. We contend that, if inadequate task sensitivity was the primary reason that performance decrements were not detected, the sleep restriction protocol should have had observable effects on other measures (i.e., the physiological and perceptual responses).

Effort sensation and RPE were also largely unaffected by sleep restriction. Previous research has noted an increase in RPE following total sleep deprivation [16] and sleep restriction [51]. However, the physical performance tasks in these investigations were of a significantly longer duration than the tasks used in the current study. Myles [52] concluded that total sleep deprivation had no effect on RPE following short-duration tasks (30 s), but that increases in RPE were observed when tasks were of a longer duration (15–50 min). The tasks in the current study were short and interspersed with rest periods, and the sleep restriction was less severe than the aforementioned protocols. The work tasks and work schedule in the current study, designed to simulate fireground work, resulted in no effect of 4 h of sleep restriction on RPE.

The literature seems to indicate that long-duration self-paced tasks may be impaired by sleep restriction [16,53], but maximal task performance can be maintained [15,16]. This suggests that task characteristics–intensity, duration, volume of active musculature, continuous or discontinuous, self-paced or externally paced–may moderate the effect of sleep restriction on physical work performance. Researchers investigating cognitive performance have concluded that the cognitive tasks most sensitive to sleep loss are long in duration, demand continuous attention, and have low predictability [54]. In addition, performance feedback has been shown to improve task performance by augmenting motivation levels in a serial reaction time task after one-night of total sleep deprivation [55]. On this basis, we suggest that the domain-specific nature of tasks may have been inherently interesting to firefighters and that the variable intensities, frequent task rotation, and repeated rest breaks may have enabled firefighters to maintain physical performance despite sleep restriction. Future research is required to investigate the impact of restricted sleep on isolated firefighting tasks performed consistently over a prolonged period (e.g., creating a fire break), and in the context of urgent, high-intensity work (e.g., protecting a home), where self-pacing may not be practicable.

Despite no between-group differences in self-paced task performance, decrements to worker activity in the sleep-restricted participants were evident during the rest periods. The between-condition difference was particularly pronounced during the rest periods; total activity counts were 20.5% greater for the control group compared to the sleep-restricted group. Given that no concurrent declines in task performance were apparent, it appears that sleep-restricted participants down-regulated their activity during periods when movement was not essential for task completion, i.e., during the rest periods provided between tasks within the physical work circuit or between circuits. It is plausible that the sleep restricted participants may have decreased their incidental activity during the rest periods. However, since activity was recorded in 1-min epochs and some of the tasks in the physical work circuit were shorter than this duration, the sampling rate did not allow more precise delineation between the work and rest components within each physical task.

It is unclear whether the sleep-restricted participants were actively adapting their behaviour, or whether reduced worker activity was observed following subconscious adaptations as a result of the fatigue induced by restricted sleep. In support of the former interpretation, competitive sporting literature suggests that the most important parameter for establishing pacing strategies is knowledge of the end-point of an activity [56,57]. In the current study, participants were aware of the demands expected of them, i.e., how many work circuits, task order, duration of task performance, and their opportunity for rest. Understanding these expectations may have allowed participants to modulate their behaviour so that the allocation of resources could be prioritised to the completion of the physical work tasks. However, if the sleep-restricted participants deliberately down-regulated their activity during the rest periods, it is not known whether this contributed to the preservation in physical task performance or simply occurred concurrently. Furthermore, we cannot conclude whether physical performance would continue to be maintained if tasks were more frequent, of higher intensity, or if the protocol was extended. Nevertheless, the activity data suggest that, despite the lack of performance differences between groups, the intervention had some influence on the sleep-restricted group. While potential explanations for this finding are discussed above, further research is required to explain how firefighters regulate their work behaviour throughout a multi-day wildfire deployment. This may involve describing the pattern of work behaviour in more detail, determining what information firefighters use to regulate their work behaviour and if these laboratory observations translate onto the fireground. This information is essential for fire agencies, especially when developing policy that informs shift lengths and the frequency of rest breaks during wildfire suppression deployments

## Conclusion

This is the first study to investigate the effects of sleep restriction on firefighters’ physical performance. Our results indicate that two nights of 4 h sleep restriction did not adversely affect firefighters’ performance of self-paced, physical work tasks. However, the sleep restricted group was less physically active, which could reflect behavioural adaptations made during rest periods, e.g., passive rest (such as sitting still and lying down) preferred over active rest activities (such as walking). Future research should examine how firefighters respond to sleep restriction over prolonged periods (> 2 nights of sleep restriction), under conditions where the sleep restriction is more severe (< 4 h per night), and when performing longer duration and/or high-intensity tasks. In addition, a greater understanding of how firefighters regulate their work behaviour patterns throughout a multi-day wildfire is essential. Further understanding of the effects of sleep restriction on firefighters’ physical performance will enable fire agencies to manage their crews for improved health, safety, and productivity on the fireground.
